# Complexes in the Photocatalytic Reaction of CO_2_ and H_2_O: Theoretical Studies

**DOI:** 10.3390/ijms11082792

**Published:** 2010-07-29

**Authors:** Dongmei Luo, Ning Zhang, Sanguo Hong, Huanwen Wu, Zhihua Liu

**Affiliations:** 1 Department of Chemistry, Nanchang University, Nanchang 330031, China; E-Mails: luodongmei1976@yahoo.com.cn (D.L.); sanguohong@jxedu.gov.cn (S.H.); wuhuanwen123@126.com (H.W.); lzh851104@yahoo.com.cn (Z.L.); 2 Department of Chemistry, QiqihaerUniversity, Qiqihaer 161006, China

**Keywords:** e–(H_2_O/CO_2_), h^+^–(H_2_O/CO_2_), photocatalytic reaction, theoretical studies

## Abstract

Complexes (H_2_O/CO_2_, e–(H_2_O/CO_2_) and h^+^–(H_2_O/CO_2_)) in the reaction system of CO_2_ photoreduction with H_2_O were researched by B3LYP and MP2 methods along with natural bond orbital (NBO) analysis. Geometries of these complexes were optimized and frequencies analysis performed. H_2_O/CO_2_ captured photo-induced electron and hole produced e–(H_2_O/CO_2_) and h^+^–(H_2_O/CO_2_), respectively. The results revealed that CO_2_ and H_2_O molecules could be activated by the photo-induced electrons and holes, and each of these complexes possessed two isomers. Due to the effect of photo-induced electrons, the bond length of C=O and H-O were lengthened, while H-O bonds were shortened, influenced by holes. The infrared (IR) adsorption frequencies of these complexes were different from that of CO_2_ and H_2_O, which might be attributed to the synergistic effect and which could not be captured experimentally.

## 1. Introduction

Greenhouse gas CO_2_ is the primary cause of global warming. The transformation from CO_2_ to fuel-like hydrocarbon is becoming one of the most important areas of chemical research, which is not only for solving the urgent greenhouse effect, but also for finding a new approach to obtain energy.

As the Earth’s ultimate power supply and an environmentally friendly energy source [1–5], light can activate reactants and promote a reaction. While the reduction of CO_2_ with H_2_O is very difficult with thermal surface catalysis, photo stimulating surface catalysis is of great theoretical and practical value. Many researches [6] have shown that CO_2_ could be reduced by water vapor or solvent with photocatalysts. Among these photocatalysts, TiO_2_ or modified TiO_2_ may promote the photoreduction of CO_2_ to useful organic compounds [7–13], such as formate, carbon monoxide, methane and ethanol. The optical and electronic properties of nanostructured TiO_2_ can be adjusted by thermal treatments [14], supported film growth [15], and metal-ion doping [16].

During our research on the reaction of H_2_O and CO_2_ over photocatalysts, we found methanol as a product [17], which meant that there must be some advantageous factors for forming C–H bond in the reaction. The photocatalytic production of CH_4_ from CO_2_ and H_2_O on Pt/SrTiO_3_ catalysts has also been reported by Hemminger *et al.* [18].

H_2_O may reduce CO_2_ to CH_4_, CH_3_OH, and CO over the anchored titanium oxide catalysts at 323 K under UV irradiation [19]. A reaction scheme is proposed for the photocatalytic reduction of CO_2_ with H_2_O as follows: ${\text{TiO}}_{2}\overset{\text{h}\nu }{\to }{\text{e}}_{\text{cb}}^{-}({\text{TiO}}_{2})+{\text{h}}_{\nu \text{b}}^{+}({\text{TiO}}_{2})$. Incident photons are absorbed by TiO_2_, and photo-excited electrons (e^-^) and positive holes (h^+^) are produced in the catalyst by charge transfer to the excited state of (Ti^3+^–O^−^)*. Furthermore, the photo-excited electrons and holes in the lattice are separated and trapped by appropriate sites of TiO_2_ to avoid recombination. The interaction of CO_2_ molecules with the excited state of (Ti^3+^–O^−^)* leads to the formation of CO_2_^•−^ radicals [5]. The semiconductor (SC) bandgap excitation leads to the formation of conduction band electrons and valance band holes, which serve as the sites for photoreduction and photooxidation, respectively [13]. The photo–oxidation of H_2_O led to the formation of hydroxyl radicals OH and H^+^ [14].

(1) $$\text{SC}\overset{h\nu >E_{g}}{\to }\text{SC }({\text{h}}_{\nu b}^{+}+{\text{e}}_{cb}^{-})$$

(2) $${\text{H}}_{2}\text{O}+{\text{h}}_{\nu b}^{+}\to {\text{H}}_{2}{\text{O}}^{+}\to •\text{OH}+{\text{H}}^{+}$$

The hydroxyl radicals generate oxygen while H^+^ ions form hydrogen by capturing conduction band electrons [15,16,18].

Photocatalytic reaction is the interaction between photo-inducted electrons and holes on the photocatalyst surface with reactants. In order to know the details of the interaction among the reactants, the photo-induced electrons and holes, and the situation of activated reactants, which were referenced by Inokuchi Y. *et al.* [20], we calculated the interaction between photo-induced electrons and holes with H_2_O/CO_2_ as complexes. There are few reports on such complexes.

## 2. Computational Details

**Figure 1:**
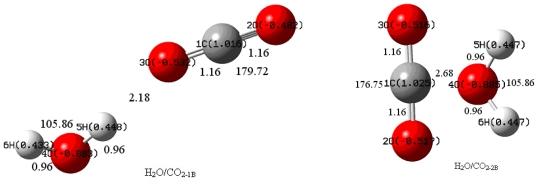

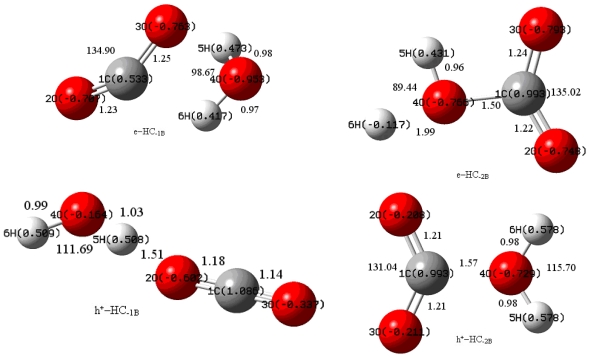
Optimized geometry structure and total NBO charge distribution of the complexes by B3LYP.

The geometries of H_2_O, CO_2_, CO_2_^•−^, H_2_O^+^, H_2_O/CO_2_, e–(H_2_O/CO_2_) and h^+^–(H_2_O/CO_2_) were respectively optimized and then they were analyzed by natural bond orbital (NBO) [21] using density functional theory (DFT) (with correcting coefficient 0.96 of vibrational frequencies) [22–25] at the B3LYP [26–29] level and Møller–Plesset (MP2 (with correcting coefficient 0.92) ) perturbation theory [30] on different basis sets. Among these basis sets, 6–311 g* [31,32] was employed in calculations as the optimal one. This work was carried out with the GAUSSIAN 03 program package [33]. CO_2_ and H_2_O were activated by photo-induced electrons and holes, which was confirmed by the change of geometry structure parameters and the charge distribution. NBO atomic charges and dipole moments of CO_2_ and H_2_O are listed in Table 1, and the geometrical parameters are shown in Figure 1. All these data indicated great changes in the optimum geometrical parameters of H_2_O/CO_2_, e–(H_2_O/CO_2_) and h^+^–(H_2_O/CO_2_) complexes. The interaction energies when the photo-induced electrons and the holes were also taken into account. The thermochemical parameters of the system are shown in Table 2.

Herein, H_2_O/CO_2-1_ refers to the complex of CO_2_ and H_2_O combined by O···H···O (probable hydrogen bond between CO_2_ and H_2_O, similarly hereinafter); H_2_O/CO_2-2_, complex of CO_2_ and H_2_O combined by C···O (probable bond between the carbon atom of CO_2_ and the oxygen atom of H_2_O, similarly hereinafter); e–HC_−1_, the complex of CO_2_ and H_2_O combined by O···H···O captured an electron; e–HC_−2_, the complex of CO_2_ and H_2_O combined by C···O captured an electron, h^+^–HC_−1_, the complex of CO_2_ and H_2_O combined by O···H···O captured a hole; (h^+^–HC_−2)_, the complex of CO_2_ and H_2_O combined by C···O captured a hole.

## 3. Results and Discussion

### 3.1. Geometric Parameters Analysis

**Figure 2:**
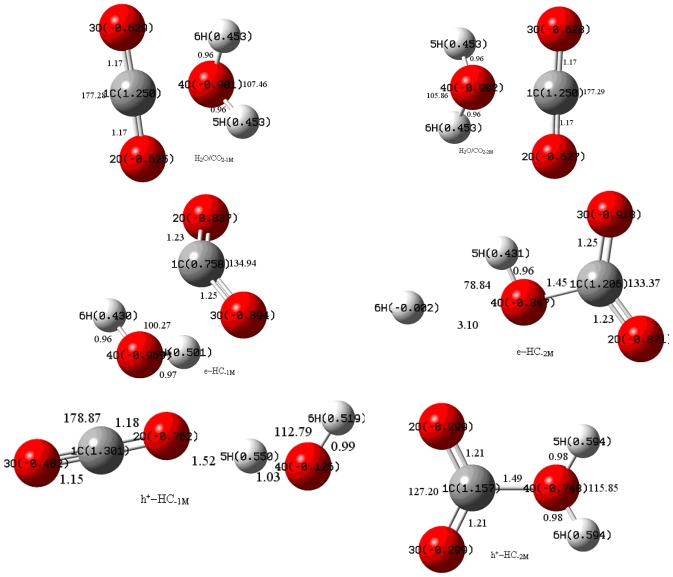
Optimized geometry structure and total NBO charge distribution of the complexes by MP2.

As shown in Figures 1 and 2, the geometrical parameters of all complexes revealed that bond lengths and bond angles were almost the same in H_2_O/CO_2-1_ and H_2_O/CO_2-2_. According to both B3LYP and MP2 method, the total dipole moments of H_2_O/CO_2_ was a little greater than that of H_2_O, indicating that the complex H_2_O/CO_2_ could not obviously change the properties of H_2_O and CO_2_ molecules.

Calculated structural parameters of e–HC_−1_ and e–HC_−2_ by B3LYP were different from that of CO_2_ and H_2_O. The C=O bond was lengthened by 0.06–0.09 Å compared to that in CO_2_ while approximately equal to that in CO_2_^•−^ and the length of H-O bond was also lengthened by 0.01–0.02 Å compared to that in H_2_O while it was shorter than that in H_2_O^+^. Therefore, CO_2_ was activated and the repulsion force between C and O was strengthened. The bond angle A_H-O-H_ was reduced by 7–17 degrees compared to that in H_2_O/CO_2_, while the bond length of H–O was 3.10 Å by MP2, which indicated the dissociation of the bond. The bond length of (CO_2_) O···H was 1.89 Å, which was close to that of hydrogen bond. The photo–induced electron in e–HC_−1_ enhanced the activation of CO_2_ and H_2_O. In e-HC_−2_, the bond length of (H_2_O) O···C was 1.50 Å by B3LYP and 1.45 Å by MP2, which was close to the length of C–O bond. One of the H atoms of H_2_O was dissociated from the original bonding O atom. In h^+^–HC_−1_, it was shown by B3LYP that one C–O bond length was shortened by 0.02 Å compared to that of CO_2_ and the other was lengthened 0.02 Å. The short one was near the triple bond, which might lead to CO formation. At the same time the O atom of CO_2_ may form a hydrogen bond with a length of 1.51 Å. The corresponding O–H bond was lengthened 0.07 Å. In h^+^–HC_−2_, both C–O bonds were lengthened 0.05 Å, C···O (H_2_O) bond length was 1.57 Å, while both H–O bonds were lengthened 0.02 Å, and the bond angle of O–H–O was increased by about 6–10 degrees. On the other hand, by MP2, the calculated structural parameters of h^+^–HC_−2_ were much different than from B3LYP, which indicated that the h^+^–HC_−1_ complex was not as stable as h^+^–HC_−2._ This was consistent with the following thermochemistry data. The dipole moments of both h^+^–HC_−1_ and h^+^–HC_−2_ were about three-times greater than that of H_2_O/CO_2-1_ and H_2_O/CO_2-2_ when calculated by B3LYP, and even greater when calculated by MP2, which indicated the breaking of symmetry in both h^+^–HC_−1_ and h^+^–HC_−2_.

### 3.2. NBO Atomic Charge Analysis

The natural electron configuration of the complexes, as shown in Table 1, revealed the population of charges in H_2_O/CO_2,_ which was different from that of H_2_O (O: 2s(1.75)2p(5.11)3d(0.01), H: 1s(0.56)) and CO_2_ (C: 2s(0.64)2p(2.31)3p(0.03)3d(0.02); O: 2s(1.72)2p(4.76)3d(0.01)). This was evidence for the formation of a London dispersion complex [34]. The natural electron configuration of CO_2_^•−^ and H_2_O^+^ were shown as follows: the natural electron configuration of the C atom was 2s(0.97)2p(2.39)3s(0.06)3p(0.04)3d(0.01) and that of the O atom was 2s(1.74)2p(5.00) by B3LYP, while 2s(0.94)2p(2.20)3s(0.05)3p(0.04)3d(0.02) of the C atom and 2s(1.75)2p(5.12)3d(0.01) of the O atom by MP2 in CO_2_^•−^ The natural electron configuration of the O atom was 2s(1.81) 2p(4.26) and that of the H atom was 1s (0.46) by B3LYP, while 2s(1.79)2p(4.28)3d(0.01) of the O atom and 1s(0.46) of the H atom by MP2 in H_2_O^+^.

The population of charge in e–(H_2_O/CO_2_) was dispersed both by B3LYP and MP2. In e-HC_−1_, there was 0.06 electrons on 3*s* orbital of C atom, 0.04 electrons on 3*p* orbital, and 0.01 electrons on 3*d* orbital by B3LYP. In e–HC_−2_, 3*s* orbital of C atom gained 0.00 electron, 0.05 (0.04 electrons by MP2) electron on 3*p* orbital and 0.02 (0.03 electron by MP2) electrons on 3*d* orbital by B3LYP. The contribution to the hybrid orbital from 2*s* orbital and 2*p* orbital were different in e–HC_−1_ and e–HC_−2_. The particular natural electron configuration of a H atom in e–HC_−2_ indicated that the H atom was a free radical which had 1.12 (1.00 electron by MP2) electrons on its 1*s* orbital, and 0.01 electrons on the 2*s* orbital of another H atom for the joining of the photo–induced electron by B3LYP.

The population of the charge in h^+^–(H_2_O/CO_2_) was dispersed. There was not so much difference in the charge distributions of h^+^–HC_−1_ and h^+^–HC_−2_ on the C atom and H atom by B3LYP, while the charge distribution of h^+^–HC_−2_ was higher than that of h^+^–HC_−1_ by MP2. The contribution of 2*p* orbital in O atom of h^+^–HC_−1_and h^+^–HC_−2_ to the hybrid orbital was different. As for H_2_O/CO_2-1_ and H_2_O/CO_2-2_, the population of the charge was almost the same by both calculating methods.

The total NBO charge distribution of the complexes is presented in Figures 1 and 2. The positive charge on C was 0.460 electrons in e–HC_−1_, which was lower than that in e–HC_−2_. The O atom of H_2_O in e–HC_−1_obtained more negative charge than that of the others in both e–HC_−1_and e–HC_−2_ H atom was negatively charged for the effect of the photo-induced electron, where the corresponding values for the isolated CO_2_^•−^ and H_2_O^+^ are shown as follows: 0.513 e^−^ for C and −0.756 e^−^ for O by B3LYP while 0.753 e^−^ for C and −0.877 e^−^ for O by MP2 in CO_2_^•−^ −0.070 e^−^ for O and 0.535 e^−^ for H by B3LYP, and −0.082 e^−^ for O and 0.541 e^−^ for H by MP2 in H_2_O_+_. It is interesting that the charge of O atom from H_2_O in h^+^–HC_−1_ was different from that in h^+^–HC_−2_ by both methods for different chemical environment around them. The unbalanced charge distribution of the two O atoms from CO_2_ in h^+^–HC_−1_ was because of the tendency to form hydrogen bond. This phenomenon also existed in H_2_O/CO_2-2_.

The total NBO charge distribution in H_2_O/CO_2-2_ was different from that of H_2_O (O: −0.870 e^−^, H: 0.435 e^−^), CO_2_ (C: 1.000 e^−^, O: −0.500 e^−^) and H_2_O/CO_2-1_ because of the interaction between C atom and O atom (from H_2_O). The greatest difference of the two calculating method is shown in e-HC_−1_and e-HC_−2_, especially for C and H atoms, while the tendency of the H dissociation was almost the same by B3LYP and MP2.

### 3.3. Thermochemistry Parameters Analysis

Thermochemistry parameters of the system are presented in Table 2. The interaction energies of CO_2_ and H_2_O molecule including photo-induced electrons and corresponding holes were calculated using the following equations:

(3) $$\Delta E_{HC}=E_{HC}-E_{{CO}_{2}}-E_{H_{2}O}$$

(4) $$\Delta E_{e-HC}=E_{e-HC}-E_{{CO}_{2}^{•-}}-E_{H_{2}O}$$

(5) $$\Delta E_{h+HC}=E_{h+HC}-E_{{CO}_{2}}-E_{H_{2}O^{+}}$$

In the equation (1), ΔE_HC_ could represent the calculated interaction energy values of the sum of electronic and thermal energies (E_tot_), the sum of electronic and thermal enthalpies (H) and the sum of electronic and thermal free energies (G) of HC_−1_ and HC_−2_. In the equation (2) Δ*E_e–HC_* represents the same items of e–HC_−1_ and e–HC_−2_, and then in the equation (3) Δ*E_h+HC_* represents that of h^+^–HC_−1_ and h^+^–HC_−2_. The corresponding entropy values were calculated by the following equation:

(6) $$\Delta S=\frac{\Delta H-\Delta G}{T}$$

T refers to the reaction temperature of our experiment 333 K [17].

H_2_O/CO_2-2_ had the maximum value of the entropy (ΔS = −128.80 J/mol·K ) for its loose structure and poor symmetry. h^+^–HC_−1_ had the highest energy (ΔE_tot_ = −84.70 KJ/mol) for the existence of dissociative H atom among the six complexes, while e–HC_−2_ had the minimum entropy (ΔS = −243.75 J/mol·K ) and the lowest energy (ΔE_tot_ = 14.54 kJ/mol) compared to the other five for its relatively stable skeletal structure as well as its good symmetry by B3LYP. This result was different from that by MP2, where HC_−1_ and H_2_O/CO_2-2_ had the maximum values of the entropy (ΔS = −264.23 J/mol·K) and h^+^–HC_−1_ had the highest energy (ΔEtot = −77.75 kJ/mol).

The enthalpy values of most of the six complexes were higher than that of the sum of CO_2_ and H_2_O, the sum of CO_2_ and H_2_O^+^, and the sum of H_2_O and CO_2_^•−^ for the forming of new bond and radicals. The Gibbs free energy values of most of the six complexes were lower than that of the sum of CO_2_ and H_2_O, the sum of CO_2_ and H_2_O^+^, and the sum of H_2_O and CO_2_^•−^, where the necessary energy for photo-induced electrons and holes to charge the reactants from the environment of the photoreaction system is available. It is interesting that the highest ΔG was in h^+^–HC_−2_, which indicated the instability of h^+^–HC_−1_. This was consistent with the structural parameters at the same level. No thermochemistry experimental data now for these complexes were available for the complicated photocatalytic reaction environment. Counterpoise calculations at the B3LYP/6-311G*//6-311++G** level and the MP2/6-311G*//6-311++G** level suggested that basis set superposition errors (BSSE) were nearly the same (2.09 kJ/mol) for all conformers. Since it is not clear that such corrections improved the reliability of the results [35–40], we did not included them in Table 2.

At the computational levels we employed, the skeletal bending vibration of CO_2_^•−^ was at 711.03 cm^−1^, the symmetrical stretching vibration and the asymmetrical stretching vibration of C=O at 1277.34 cm^−1^ and 1621.78 cm^−1^ respectively by B3LYP, while the corresponding absorption frequencies of CO_2_^•−^ by MP2 were at 689.17 cm^−1^, 1232.93 cm^−1^ and 1613.98 cm^−1^, respectively.

**Figure 3:**
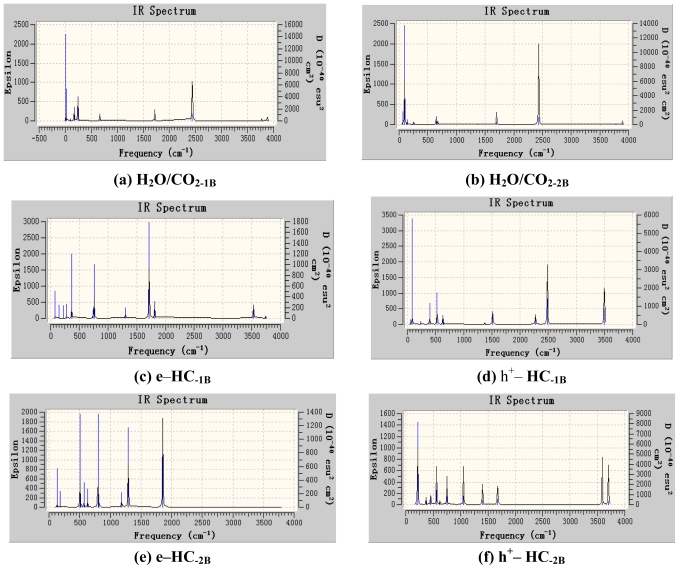
IR Spectrum of the complexes at B3LYP/6–311G* Level (GV 4). **(a)** H_2_O/CO_2-1B_; **(b)** H_2_O/CO_2-2B_; **(c)** e–HC_−1B_; **(d)** h^+^– HC_−1B_; **(e)** e–HC_−2B_; **(f)** h^+^– HC_−2B_.

**Figure 4:**
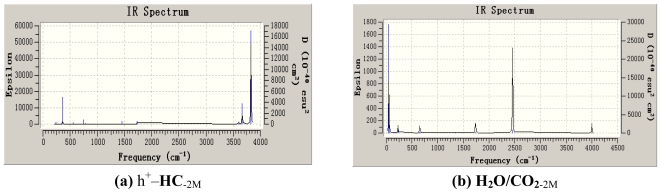

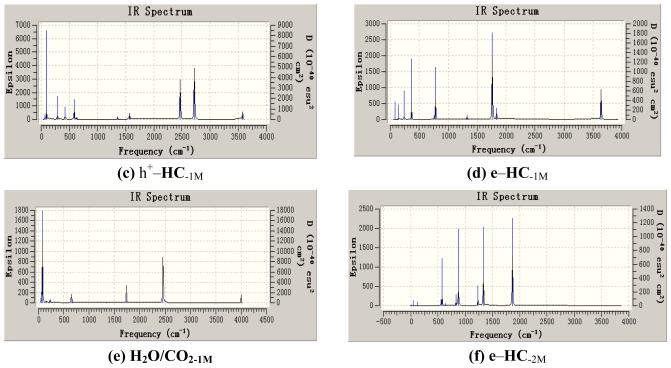
IR Spectrum of the complexes at MP2/6–311G* Level (GV 4). **(a)** h^+^–HC_−2M_; **(b)** H_2_O/CO_2-2M_; **(c)** h^+^–HC_−1M_; **(d)** e–HC_−1M_; **(e)** H_2_O/CO_2-1M_; **(f)** e–HC_−2M._

The infrared absorption spectra of complexes herein became more complicated for the interaction between H_2_O and CO_2_ and the effect of photo-induced electrons and holes as shown in Figures 3 and 4. In H_2_O/CO_2-1_, the asymmetrical stretching vibration of O–C≡O at 2342.28 cm^−1^ by B3LYP and 2261.42 cm^−1^ by MP2, respectively.

There was no different infrared absorption between H_2_O/CO_2-1_ and H_2_O/CO_2-2_ by MP2. In e–HC_−1_, the stretching vibration of H–O (O–H···O=C) was at 3390.72 cm_−_^1^ by B3LYP, and the other stretching vibration of H–O at 3590.40 cm^−1^, the scissoring rocking vibration of C=O at 710.40 cm^−1^, the symmetrical stretching vibration and the asymmetrical stretching vibration of C=O at 1253.76 cm^−1^ and 1648.32 cm^−1^, respectively. By MP2, the infrared absorption frequencies of e–HC^−1^ were somewhat red shifted. In e–HC_−2_, the stretching vibration of C···O was at 546.72 cm^−1^ by B3LYP, the symmetrical stretching vibration and the asymmetrical stretching vibration of C=O at 1242.24 cm^−1^ and 1782.72cm^−1^, respectively. There are many differences of which by MP2, the skeletal rocking vibration at 513.47 cm^−1^, the in-plane rocking vibration and the out-of-plane rocking vibration of H–O at 1137.10 cm^−1^ and 531.10 cm^−1^, respectively, the stretching vibration of H–O at 3525.12 cm^−1^, the in-plane bending vibration and the out-of-plane bending vibration of C=O at 765.76cm^−1^ and 805.19cm^−1^, the symmetrical stretching vibration and the asymmetrical stretching vibration of C=O at 1225.77 cm^−1^ and 1716.58 cm^−1^, the stretching vibration of C···O at 587.59 cm^−1^.

There are some synergistic effects in h^+^–HC_−1_ by B3LYP. The symmetrical stretching vibration of O···H–O was at 2182.68 cm^−1^, the out-of-plane bending vibration and the in-plane bending vibration of O–C≡O at 603.19 cm^−1^ and 612.05 cm^−1^, respectively, the symmetrical stretching vibration of O–C≡O at 1322.16 cm^−1^, the synergistic effect of the asymmetrical stretching vibration of O···H–O and O–C≡O at 2384.96 cm^−1^. While by MP2 in h^+^–HC_−1_, the out-of-plane bending vibration of O···H–O was at 538.36 cm^−1^, the symmetrical stretching vibration and asymmetrical stretching vibration of O–C≡O at 1356.56 cm^−1^ and 2275.21 cm^−1^. The asymmetrical stretching vibration of O···H–O was at 2507.93 cm^−1^, the stretching vibration of H–O at 3309.60 cm^−1^. In h^+^– HC_−2_ by B3LYP the in-plane bending vibration of C=O was at 540.22 cm^−1^, the stretching vibration of C···O at 601.69 cm^−1^, and the synergistic effect of the asymmetrical stretching vibration of C=O at 722.74 cm^−1^, the asymmetrical stretching vibration of H···O=C at 1012.45 cm^−1^. While by MP2 the stretching vibration of C···O at 685.57 cm^−1^, the scissoring rocking vibration of C=O at 511.11 cm^−1^, and the symmetrical stretching vibration of C=O at 1334.48 cm^−1^. It indicated that both the two calculating method revealed the unreasonable data to h^+^–HC_−2_, therefore the complex was unstable, therefore h^+^–(H_2_O/CO_2_) had only one stable structure h^+^–HC_−1_.

## 4. Conclusion

The charge distribution of H_2_O and CO_2_ was changed because of the joining of the photo-induced electrons and holes both by MP2 and by B3LYP. The effect from photo-induced electrons and holes in complexes activated H_2_O and CO_2_ herein. H_2_O has been known to exist as –OH group on the surface of catalysts by our experiment and the literature [12]. H_2_O was activated for the lengthened O–H bond in h^+^–(H_2_O/CO_2_). The possible hydrogen bond and CO in h^+^–(H_2_O/CO_2_) indicated that the hole was advantageous during the photoreduction of CO_2_ by H_2_O. The IR absorption of e–(H_2_O/CO_2_) and h^+^–(H_2_O/CO_2_) could not be captured experimentally, and some synergistic effect appeared in IR adsorption spectra which were consistent with the state of complexes and their geometric parameters, therefore the result of calculations revealed useful information for understanding the reaction system.

The results of calculations indicated that the action of photo-induced electrons and holes to H_2_O/CO_2_ was stronger than the interaction between H_2_O and CO_2,_ therefore CO_2_ was activated in e–(H_2_O/CO_2_) and h^+^–(H_2_O/CO_2_), the bond length of R_O=C_ lengthened in e-(H_2_O/CO_2_), the charge on C and O changed greatly, the density of electron cloud on C strengthened in e–(H_2_O/CO_2_), and the probability for C to combine with –OH or H increased, which might be favorable for forming CH_3_OH.

## Figures and Tables

**Table 1:** Natural Electron Configuration of the complexes.

M	E	h^+^–HC_−1_	h^+^–HC_−2_	e-HC_−1_	e-HC_−2_	H_2_O/CO_2-1_	H_2_O/CO_2-2_
B	O	2s(1.69) g	2s(1.75)	2s(1.74)	2s(1.72)	2s(1.72)	2s(1.72)
3		2p(4.90) h	2p(4.45)	2p(4.96)	2p(5.02)	2p(4.75)	2p(4.78)
L		3d(0.01) i	3d(0.01)	3d(0.01)	3d(0.01)	3d( 0.01)	3d( 0.01)
Y	C	2s(0.62)	2s(0.68)	2s(0.96)	2s(0.67)	2s(0.64)	2s(0.64)
P		2p(2.25)	2p(2.27)	2p(2.40)	2p(2.27)	2p(2.30)	2p(2.29)
		3p(0.02)	3p(0.03)	3s(0.06)	3p(0.05)	3p(0.03)	3p(0.03)
		3d(0.02)	3d(0.02)	3p(0.04)	3d(0.02)	3d(0.02)	3d(0.02)
B				3d(0.01)			
	
3	O	2s(1.72)	2s(1.75)	2s(1.75)	2s(1.73)	2s(1.72)	2s(1.72)
L		2p(4.60)	2p(4.45)	2p(5.01)	2p(5.05)	2p(4.80)	2p(4.78)
Y		3d(0.01)	3d(0.01)	3d( 0.01)	3d(0.01)	3d(0.01)	3d(0.01)
P	H	1s( 0.49)	1s(0.42)	1s(0.52)	1s(0.56)	1s(0.55)	1s(0.55)
				2s(0.01)	2s(0.01)		
	
B	O	2s(1.79)	2s(1.65)	2s(1.75)	2s(1.75)	2s(1.75)	2s(1.75)
3		2p(4.37)	2p(5.07)	2p(5.19)	2p(5.00)	2p(5.13)	2p(5.13)
L		3d( 0.01)		3d(0.01)		3d( 0.01)	3d(0.01)
Y	H	1s( 0.49)	1s(0.42)	1s(0.52)	1s(1.12)	1s( 0.56)	1s(0.55)
P				2s(0.01)			
	
M	O	2s(1.70)	2s(1.75)	2s(1.74)	2s(1.73)	2s(1.72)	2s(1.72)
P		2p(5.05)	2p(4.54)	2p(5.08)	2p(5.13)	2p(4.89)	2p(4.89)
2				3p(0.01)			
		3d(0.01)	3d(0.01)	3d(0.01)	3d(0.01)	3d( 0.01)	3d( 0.01)
M	C	2s(0.63)	2s(0.66)	2s(0.93)	2s(0.66)	2s(0.65)	2s(0.65)
P		2p(2.02)	2p(2.11)	2p(2.20)	2p(2.06)	2p(2.05)	2p(2.05)
2		3p(0.02)	3p(0.03)	3s(0.05)	3p(0.04)	3p(0.03)	3p(0.03)
		3d(0.03)	3d(0.03)	3p(0.04)	3d(0.03)	3d(0.03)	3d(0.03)
M				3d(0.02)			
	
P	O	2s(1.72)	2s(1.75)	2s(1.75)	2s(1.74)	2s(1.72)	2s(1.72)
2		2p(4.75)	2p(4.54)	2p(5.13)	2p(5.17)	2p(4.89)	2p(4.89)
		3d(0.01)	3d(0.01)	3d( 0.01)	3d(0.01)	d(0.01)	33d(0.01)
M	H	1s( 0.45)	1s(0.40)	1s(0.49)	1s(0.56)	1s(0.54)	1s(0.54)
P							
	
2	O	2s(1.78)	2s(1.64)	2s(1.75)	2s(1.73)	2s(1.74)	2s(1.74)
		2p(4.34)	2p(5.11)	2p(5.20)	2p(5.10)	2p(5.16)	2p(5.16)
M		3d( 0.01)		3d(0.01)	3d(0.01)	3d( 0.01)	3d(0.01)
P	H	1s( 0.48)	1s(0.40)	1s(0.57)	1s(1.00)	1s( 0.54)	1s(0.54)
2				2s(0.01)			

**Table 2:** Thermochemistry Parameters of Complexes.

Method parameter	B3LYP				MP2			
	ΔE_tot_ kJ/mol	ΔH kJ/mol	ΔG kJ/mol	ΔS J/mol·K	ΔE_tot_ kJ/mol	ΔH kJ/mol	ΔG kJ/mol	ΔS J/mol·K
**HC**_−_**1**	4.68	0.28	53.78	−160.66	−26.26	−26.26	52.52	−264.32
**HC**_−_**2**	−2.48	−7.44	35.45	−128.81	−26.26	−26.26	52.52	−264.32
**e-HC**_−_**1**	−69.04	−74.00	−4.80	−207.81	−73.51	−68.26	2.62	−237.73
**e-HC**_−_**2**	14.54	9.58	90.75	−243.75	−28.88	−23.63	39.38	−211.34
**h^+^–HC**_−_**1**	−84.70	−93.49	−37.77	−167.33	−77.75	−58.17	−8.13	−167.83
**h^+^–HC**_−_**2**	−25.74	−30.68	36.74	−202.46	42.47	47.24	108.29	−204.76
